# The EmpaTeach intervention for reducing physical violence from teachers to students in Nyarugusu Refugee Camp: A cluster-randomised controlled trial

**DOI:** 10.1371/journal.pmed.1003808

**Published:** 2021-10-04

**Authors:** Camilla Fabbri, Katherine Rodrigues, Baptiste Leurent, Elizabeth Allen, Mary Qiu, Martin Zuakulu, Dennis Nombo, Michael Kaemingk, Alexandra De Filippo, Gerard Torrats-Espinosa, Elizabeth Shayo, Vivien Barongo, Giulia Greco, Wietse Tol, Karen M. Devries

**Affiliations:** 1 London School of Hygiene & Tropical Medicine, London, United Kingdom; 2 International Rescue Committee, New York, New York, United States of America; 3 Innovations for Poverty Action, Dar es Salaam, Tanzania; 4 Behavioral Insights Team, New York, New York, United States of America; 5 Columbia University, New York, New York, United States of America; 6 National Institute for Medical Research, Dar es Salaam, Tanzania; 7 University of Copenhagen, Copenhagen, Denmark; Emory University, UNITED STATES

## Abstract

**Background:**

School-based violence prevention interventions offer enormous potential to reduce children’s experience of violence perpetrated by teachers, but few have been rigorously evaluated globally and, to the best of our knowledge, none in humanitarian settings. We tested whether the EmpaTeach intervention could reduce physical violence from teachers to students in Nyarugusu Refugee Camp, Tanzania.

**Methods and findings:**

We conducted a 2-arm cluster-randomised controlled trial with parallel assignment. A complete sample of all 27 primary and secondary schools in Nyarugusu Refugee Camp were approached and agreed to participate in the study. Eligible students and teachers participated in cross-sectional baseline, midline, and endline surveys in November/December 2018, May/June 2019, and January/February 2020, respectively. Fourteen schools were randomly assigned to receive a violence prevention intervention targeted at teachers implemented in January–March 2019; 13 formed a wait-list control group. The EmpaTeach intervention used empathy-building exercises and group work to equip teachers with self-regulation, alternative discipline techniques, and classroom management strategies. Allocation was not concealed due to the nature of the intervention. The primary outcome was students’ self-reported experience of physical violence from teachers, assessed at midline using a modified version of the ISPCAN Child Abuse Screening Tool–Child Institutional. Secondary outcomes included student reports of emotional violence, depressive symptoms, and school attendance. Analyses were by intention to treat, using generalised estimating equations adjusted for stratification factors. No schools left the study. In total, 1,493 of the 1,866 (80%) randomly sampled students approached for participation took part in the baseline survey; at baseline 54.1% of students reported past-week physical violence from school staff. In total, 1,619 of 1,978 students (81.9%) took part in the midline survey, and 1,617 of 2,032 students (79.6%) participated at endline. Prevalence of past-week violence at midline was not statistically different in intervention (408 of 839 students, 48.6%) and control schools (412 of 777 students, 53.0%; risk ratio = 0.91, 95% CI 0.80 to 1.02, *p* = 0.106). No effect was detected on secondary outcomes. A camp-wide educational policy change during intervention implementation resulted in 14.7% of teachers in the intervention arm receiving a compressed version of the intervention, but exploratory analyses showed no difference in our primary outcome by school-level adherence to the intervention. Main study limitations included the small number of schools in the camp, which limited statistical power to detect small differences between intervention and control groups. We also did not assess the test–retest reliability of our outcome measures, and interviewers were unmasked to intervention allocation.

**Conclusions:**

There was no evidence that the EmpaTeach intervention effectively reduced physical violence from teachers towards primary or secondary school students in Nyarugusu Refugee Camp. Further research is needed to develop and test interventions to prevent teacher violence in humanitarian settings.

**Trial registration:**

clinicaltrials.gov (NCT03745573)

## Abstract 2

In a cluster-randomised controlled trial, Dr. Camilla Fabbria and colleagues investigate whether the EmpaTeach intervention could reduce physical violence from teachers to students in Nyarugusu Refugee Camp, Tanzania.

## Author summary

**Why was this study done?:**

- Violence in and around schools is widespread, and teachers are among the most frequent perpetrators of violence against students.
- In humanitarian settings, widespread insecurity and students’ and teachers’ histories of trauma may exacerbate the risk of violence.
- Despite this, few interventions to prevent violence against children from teachers have been rigorously tested in randomised controlled trials, and none to our knowledge in humanitarian settings.

**What did the researchers do and find?:**

- We conducted a cluster-randomised controlled trial to test a brief school-based peer-led behavioural intervention to reduce teachers’ use of physical and emotional violence against students in Nyarugusu Refugee Camp, Tanzania.
- The intervention consisted of 12 sessions delivered over 10 weeks, covering empathy-building exercises and group work to learn and practice self-regulation techniques, positive disciplinary methods, and strategies to promote well-being.
- We found no evidence that the intervention reduced levels of violence at 2 and 10 months after the intervention implementation.

**What do these findings mean?:**

- We did not find evidence that the intervention was effective in improving children’s experiences of physical or emotional violence, and this could be due to a combination of design, delivery, and contextual factors.
- Further research is needed to test what intervention components and delivery strategies can be effective to reduce school-based violence in humanitarian settings.

## Introduction

More than 1 billion children experience physical, sexual, or emotional violence each year [1]. For the 33 million children estimated to be forcibly displaced by war and conflict globally as of 2019 [2], levels of violence are likely to be even higher, as several features of humanitarian crises are known risk factors [3]. These include widespread economic and social insecurity, weakened social ties and alterations to traditional household composition, threats to basic livelihoods and individual freedoms, and the breakdown of protective systems and service provision [4]. In Nyarugusu Refugee Camp, where this study takes place, violence in schools and on the way to and from schools is perceived as prevalent [5]. Norms that promote the use of violence as a way of disciplining students and supporting student learning are widespread in the camp [6], and although the majority of students report feeling safe in school, the ‘stick’ and other forms of corporal punishment are frequently used to manage large classrooms [5]. Violence in childhood and adolescence is associated with future depression, suicide attempts, violence victimisation and perpetration, and poor educational outcomes [7–10], and prevention of violence is a focus of Sustainable Development Goals 5, 8, and 16.

Schools can be a main site of exposure to violence, from peers and from school staff [11–14], but also offer tremendous opportunities for primary prevention of violence [15]. Particularly for displaced children, schools may offer some sense of normality in very challenging conditions and provide an opportunity to build aspirations and recover from trauma [16]. WHO recommends school-based approaches to improve a range of child and adolescent health outcomes [17,18], but only a handful of evaluations of programmes to prevent violence from teachers to students have been conducted globally [19,20] and, to the best of our knowledge, none in humanitarian settings. Two interventions have been rigorously trialled, and both were proven effective in reducing violence from school staff to students [21,22]; however, both programmes were implemented in low-resource but stable contexts in Uganda and Jamaica. One is a whole-school approach focused on creating a conducive operational culture in schools and encouraging behaviour change for teachers, students, and school administration over 18 months [22]. The other is focused on teacher skill development and behaviour change over 8 months, with a supported process for teachers to learn positive reinforcement, classroom management strategies, and proactive approaches to prevent child misbehaviours [21].

While corporal punishment often occurs as a normalised behaviour, it can also occur impulsively rather than instrumentally [23,24] and can be exacerbated by perceived stress [25]. Stress and other life events that generate negative affect and emotional distress can be triggers of violent behaviours and aggression [26,27]. In contexts such as humanitarian crises, characterised by high levels of stress, overcrowding, and severe resource constraints, it is plausible that teachers’ psychological well-being may be compromised and their emotion regulation weakened, resulting in increased risk of impulsive violent behaviours. To date, no specific interventions focusing on reducing teachers’ impulsive use of violence have been tested, but correlational studies suggest that integrating content on self-regulation and coping, along with positive discipline methodologies, into teacher training could be an effective mechanism to reduce violent discipline [25].

In this study, we aimed to determine whether a short peer-led intervention focused on reducing impulsive violence—known as EmpaTeach—reduced physical violence from school staff to students in Nyarugusu Refugee Camp, Tanzania, using a cluster-randomised controlled trial. EmpaTeach takes a psychological approach, aiming to improve teachers’ self-efficacy, self-regulation, and empathy for students, and to reduce teachers’ stress levels. It provides teachers with information about alternative disciplinary methods and positive classroom management strategies, and creates social support for skill development. The intervention is implemented by trained peer teachers over a 10-week period, and is thus feasible to deliver in highly resource-constrained contexts, including emergency settings.

## Methods

The Preventing Violence Against Children in Schools (PVACS) study consists of a cluster-randomised controlled trial, a parallel qualitative study, an economic evaluation, and a process evaluation. The study was approved by the London School of Hygiene & Tropical Medicine Ethics Committee (ref. 16000) and the Tanzania National Institute for Medical Research (ref. NIMR/HQ/R.8a/Vol.IX/2920). Our protocol is registered at ClinicalTrials.gov (NCT03745573) and is published elsewhere [28]. This paper describes our main trial results and is reported as per the Consolidated Standards of Reporting Trials (CONSORT) guideline (S1 CONSORT Checklist).

### Setting

Nyarugusu Refugee Camp was formed in 1996. It hosts about 80,000 Congolese refugees, some of whom have been there since the camp’s establishment, and about 60,000 Burundian refugees, who have been arriving since 2015. The camp is operated by the United Nations High Commission for Refugees and the Tanzania Ministry of Home Affairs, and the International Rescue Committee (IRC) provides all education and gender-based violence response services and, at the time of the trial, provided all child protection services.

Children attend 1 of 27 schools in the camp, which teach either a Burundian or Congolese curriculum. A 2018 survey across Nyarugusu and 2 smaller camps in the Kigoma region showed only 56% of school-aged children were enrolled. However, 78% of Congolese children in Nyarugusu attended school, owing to the protracted displacement of the Congolese population [5]. Schools in the camp face numerous challenges, including poor teacher attendance and shortages of qualified teachers. In Nyarugusu, in 2017–2018, there was a teacher to pupil ratio of up to 1:200 in primary schools; teachers received low pay and often needed to engage in other income-generating activities [5].

### Study design and participants

We conducted a 2-arm superiority cluster-randomised controlled trial with parallel assignment between November 2018 and February 2021. A cluster design was appropriate because the intervention was delivered to all teachers within a given school. A complete sample of all 27 primary and secondary schools in the camp was invited to participate All schools agreed to participate and were randomised to the intervention or to act as controls.

Allowing for a loss to follow-up of 2 schools per arm, assuming 50 interviews with students per school, a prevalence of past-week physical violence of 50%, and an intra-cluster correlation coefficient of 0.10 [22], we had 80% power to detect a 19% difference in the prevalence of our primary outcome between the intervention and control arms with a 2-sided significance level of 5%.

Three rounds of cross-sectional surveys were conducted in schools: baseline (from 19 November 2018 to 8 December 2018), midline (from 15 May 2019 to 7 June 2019, 2 months after the end of the intervention), and endline (from 23 January 2020 to 21 February 2020, 10 months after the end of the intervention). A cross-sectional design was selected rather than a cohort design to avoid issues related to attrition of individual students over time, as there was some likelihood of repatriation of Burundian refugees over the course of the study. Headteachers provided consent for data collection and intervention implementation in schools, and consent to approach individual students. Students provided informed assent, and individual teachers provided informed consent. Parental consent for children’s participation in this study was not required by relevant ethics committees. In initial consultations with camp stakeholders, it was felt that parents in this setting would prefer headteachers to consent for children in school as they had full responsibility for students during school hours, and that seeking active parental consent would preclude large numbers of children from participating, given high numbers of unaccompanied youth in the camp. Up-to-date lists of all teachers and students in grade 2 and above were obtained from IRC’s education team. These lists formed the sampling frame for the study, and a stratified random sample of 60 students per school was selected to ensure representation from different age groups. All students who could speak Kiswahili or Kirundi and who were deemed by interviewers to understand the consent procedures were eligible.

We collected survey data from students in grade 2 or above and aged 9 years and over, as they were able to respond to questions in survey format in our pre-testing. A simple random sample of school staff was invited to provide informed consent for their participation in survey data collection. At least 2 repeat visits were made to find students and staff who were sampled but absent on the day of the survey. The sample is thus intended to be representative of teachers and of students in grade 2 or above and age 9 years and over attending school in the camp.

Survey data were collected by interviewers recruited from the camp population, who received 2 weeks of intensive training before each data collection round. Interviewers read questions and response options to participants from tablet computers. Algorithms were in place to prevent accidental skipping of questions, and referrals for those who disclosed abuse (see below) were automatically triggered based on survey responses. Backchecks and data quality audits were conducted on 10% of the sample at each round of data collection by experienced interviewers who acted independently from the core field team and who reported directly to the local field coordinator. Backchecks were used to assess enumerator performance, identify errors in survey programming, and record discrepancies to address during data cleaning.

### Randomisation and masking

To ensure balance across arms, schools were stratified according to whether they served a Congolese or Burundian population, and were primary or secondary schools. An allocation list was generated by EA with a computer random number generator and an algorithm in Stata (version 15 [29]). Allocation took place at a public meeting where a representative of each school within each stratum was invited to place the name of their school in an opaque bag. A nominated person from each stratum then withdrew names from the bag, and schools were allocated either to receive the intervention or to the control condition in the sequence on the allocation list, recorded by MZ. The intervention is behavioural in nature, and it was not possible to mask participants to allocation. Similarly, allocation was not discussed with interviewers tasked with collecting survey data, but it is reasonable to assume that they could determine allocation. The statistician (BL) was masked to allocation when performing the main trial analyses. Some secondary analyses (e.g., adherence) were performed after unmasking. EA, MZ, and BL are authors and members of the PVACS research team.

### Intervention

#### Content

The EmpaTeach intervention is a behaviourally informed, self-guided teacher training intervention designed to reduce and prevent teachers’ use of corporal punishment in the classroom [30]. The content of the intervention is focused on empathy-building exercises and on group work to learn and practice self-regulation techniques, strategies to promote well-being, positive disciplinary methods, and classroom management strategies. To create intervention materials and exercises around empathy building, alternative discipline, and other ingredients, the intervention designers drew on existing programmes and also created new content [31–33]. Box 1 describes implementation in the trial [34]. The intervention theory of change is illustrated in S1 Fig.

**Box 1. Description of the EmpaTeach intervention**

**Overview**

The project targets teachers’ attitudes, beliefs, and behaviours. Teachers go through self-guided group sessions inspired by cognitive behavioural therapy—an approach that has been effectively applied to many problems, from reducing destructive behaviours to improving well-being—to help teachers challenge thinking and patterns of behaviour related to using violence as a form of punishment.

**Content and materials**

The intervention was based on a booklet developed specifically to self-guide teachers through each of the 12 sessions in the programme (developed by the Behavioural Insights Team [BIT] and IRC in English, and translated into Kiswahili and Kirundi). The booklet contained learning materials for all sessions and space to commit to practicing new strategies and record reflections on their homework assignments, which primarily consisted of home and classroom practice of the intervention techniques. For 6 of the sessions, there were accompanying videos that were produced locally as part of the intervention. Two of the sessions involved playing an interactive game with playing cards to help teachers apply learned concepts. Specifically, throughout the intervention, teachers engaged in a series of value-affirmation and empathy-building exercises based on self-reflection, and they received information about alternative disciplinary techniques (including de-escalation strategies and techniques to reward positive behaviours) and emotional regulation tools inspired by cognitive behavioural therapy to identify triggers for impulsive reactions. The intervention supported teachers in creating action plans for change for responding to students’ positive and negative behaviours and reflecting on future actions when they would encounter problems. Finally, the intervention generated social support through the group setting so that teachers could count of peers for support and advice throughout the change process. The intervention material was designed to be suitable for application with children of different age groups. The description of each classroom strategy illustrated in the booklet included adaptations for older children, and gender considerations.

A shared tablet computer was required for each group to view the videos during the sessions. Session 5, which involved learning how to co-create classroom rules with students, required a large piece of posterboard paper, a marker, and tape or glue to affix the paper to the wall. All teachers were served lunch during the introductory meeting and the programme ending party; however, neither teachers nor group coordinators received any form of payment for their participation in the intervention. Guides were provided for the coordinators of the sessions, which contained the agenda and content for each meeting.

**Procedures**

A BIT programme developer, IRC education technical unit staff, and local refugee incentive workers provided a 3-day training to 85 teachers who were nominated as group coordinators by their peers (as well as a separate 1-day training with school headteachers and discipline teachers). Day 1 was focused on facilitation skill building, and beginning to review the facilitator roles and responsibilities and the programmatic themes. On day 2, facilitators finished reviewing the programme themes and roles and responsibilities. On day 3, facilitators were split into smaller groups and practiced facilitation of sessions. The group facilitators were trained in 3 separate groups: 2 groups of Congolese facilitators and 1 group of Burundian facilitators. During the training itself, teachers participated in smaller group work activities. There were 2 trainers (1 English speaker and 1 native Kiswahili or Kirundi speaker) at each of the group facilitator trainings. As such, the trainer:teacher ratio was 2:27 to 2:30, depending on the group. These training sessions provided participants with an overview of the intervention and their roles and responsibilities, built group facilitation skills, provided a space for practicing facilitation skills and learning from other participants, prepared facilitators for potential challenges that could arise during the intervention, and built their buy-in. The coordinator training contained a review of the main lessons from each programme module, facilitator roles and responsibilities, and facilitation and organisation skills, including practice sessions facilitating mock sessions. Group coordinators did not receive additional facilitation support during the intervention implementation period, as the curriculum is intended to be self-guided. The selection of teacher facilitators followed a staged approach: First, teachers were asked to nominate peers they admired or learned from. Second, nominated teachers completed a survey about their attitudes towards the use of corporal punishment. Teachers who expressed supportive views towards the use of harsh discipline were removed from the potential facilitator cohort. Third, among the remaining teachers, those who were most proficient in facilitation skills were selected to be facilitators, and the remaining teachers became ‘back-up facilitators’.

Each group coordinator facilitated the programme in a face-to-face fashion with a group of 3–15 teachers. The first 4 sessions were condensed into two 4-hour sessions delivered over 2 weekend days. (This resulted in there being 12 sessions in total, instead of the 14 sessions specified in the PVACS study protocol.) The remainder of the sessions were held weekly until the end of the programme, and lasted about 1–1.5 hours each—with the exception of weeks 5 and 11, when groups met a second time during the week to further engage with the techniques they had learned by playing an interactive learning game. The teachers did homework each week, taking about 30 minutes. They also received 2 SMS texts per week from their group facilitators to reinforce aspects of the group sessions or homework. Each group self-determined when and where it met each week.

Each session started with a review of the previous week’s session, reflection on key concepts, and sharing of homework, including any challenges encountered. This was followed by an introduction of a short slogan capturing the main learning of the session. Then participants engaged in a series of stories that illustrated a hypothetical but common classroom situation and reflection activities, and presentation and discussion of simple classroom management and self-regulation activities they could use, followed by homework that allowed real-world practice of new techniques in teachers’ own classrooms.

**Modifications**

During programme implementation, some group coordinators expressed being unclear about how to facilitate the card games with teachers. In light of this, IRC staff offered a 1-day refresher training on the teacher card games specifically.

A number of teachers were made redundant (i.e., laid off) during the implementation of the intervention and were subsequently re-hired within weeks. This disrupted implementation of EmpaTeach across a number of teacher groups. As a result, IRC’s programme team had to re-assemble some of the intervention groups and make plans to allow for the implementation of the intervention within a shorter period of time. Nine out of 77 groups received a compressed version of EmpaTeach over a 6-week period (instead of 10 weeks).

#### Intervention development and pilot trial

The intervention design was informed by qualitative interviews and focus group discussions with teachers, school administrative staff, students, parents, and humanitarian frontline workers in the camp, and developed via iterative co-creation sessions with teachers. The intervention was intentionally designed to be short, with limited ongoing support for implementation, and focused primarily on teachers so that it could be implemented in humanitarian contexts with highly mobile populations and significant resource constraints. Teachers were selected mainly because they were seen as credible, relatable messengers with relevant experience, helping to dispel possible perceptions among teachers that the intervention was being externally imposed.

High levels of stress were reported by teachers in qualitative interviews conducted during the formative research stage, resulting from overcrowded classrooms and poor resources, as well as the teachers’ own experiences as forcibly displaced persons. The designers thus included techniques inspired by cognitive behavioural therapy focused on enhancing teacher well-being, empathy, and emotional self-regulation, which the designers hypothesized would lead to a reduction of stress-induced or impulsive uses of corporal punishment. A rapid pilot trial aimed at identifying effective behavioural strategies to shift attitudes was conducted in 2018; results showed that an empathy-building approach was the most promising in changing teachers’ attitudes about violence, compared to rights-based and information-based approaches [35].

The designers also hypothesized that teachers’ lack of training in alternative disciplinary techniques would be a barrier to reducing use of violent discipline. The initial third of the EmpaTeach curriculum was therefore dedicated to teaching and practicing positive discipline strategies and classroom management skills. This content was co-created and validated with refugee teachers through a series of iterative sessions where teachers provided feedback on the clarity of exercises, examples of local positive discipline strategies, and scenarios and stories relevant to the camp cultural context. During co-creation sessions, teachers became defensive of, and expressed support for, use of corporal punishment and violent discipline when the topic was broached. The designers thus determined that directly addressing norms on the acceptability of violence would have required highly trained facilitators to avoid inadvertent reinforcement of norms. Such facilitators are not available in the camp setting. Therefore, the intervention did not directly discourage the use of physical violence for classroom management, but instead encouraged teachers to adopt new and more effective behavioural strategies.

In 2018, a controlled before-and-after pilot study was conducted, where the whole intervention was implemented in 4 schools in a nearby refugee camp. The pilot study suggested that the intervention was acceptable to participants and feasible to deliver, and preliminary estimates indicated that the direction of effect would be towards a reduction in teachers’ use of physical violence [30].

#### Delivery

All teachers in intervention schools were invited to participate in EmpaTeach, and were assigned to a group composed of other teachers from their school. While participation was not mandatory, it was strongly encouraged and communicated as expected by school administrators. All students in each school thus had the potential to be exposed to the intervention. Groups met 12 times, for two 4-hour sessions and ten 1- to 1.5-hour sessions, which were led by peer teachers (group facilitators) guided by a tailored booklet featuring stories, lessons, and exercises. Teachers received information on positive discipline, as well as planning exercises and reinforcement SMS texts. Given that the intervention was implemented in a group setting, teachers also received social support from peers to change their behaviours. Teachers had the opportunity to discuss their experiences and challenges in the group sessions. The groups met over a 10-week period as originally intended (‘original version’) or over a 6-week period (‘compressed version’). Reasons for this are described in the Results.

Schools in the control arm did not receive any specific intervention related to violence prevention during the study.

### Harms

The intervention is behavioural in nature, and we anticipated that this would result in minimal risk for participants. We did speculate that for teachers who had experienced recent trauma, some of the empathy and reflection exercises may have resulted in increased emotional distress; however, we did not record increased levels of distress among teachers in the intervention group. All teachers were given information about where they could seek psychological support. At endline, IRC’s programme team installed a letterbox in a central location within the camp to allow for anonymous complaints and feedback on the study procedures. All teachers were informed about the existence of the letterbox during consent procedures, but no complaints were reported.

We did not expect any adverse effects of the intervention among children in intervention schools, but anticipated that during survey data collection, children would disclose experiences of abuse that would necessitate referral to child protective services. During intervention implementation, IRC’s child protection team made regular visits to study schools as part of their routine activities and offered routine support and safeguarding services to students. During research activities, children were informed during the consent process that we would need to pass their details on to child protection officers if they disclosed information that made the research team think that ‘their safety or well-being might have been at risk or that they had been hurt’. The student survey contained a number of algorithms based on students’ age and responses to survey questions that automatically generated different interview finishes that prompted enumerators to follow recommended referral pathways. Specific disclosures about forms and time frames of abuse that would necessitate referrals were based on predefined criteria agreed on with IRC’s child protection team. Teachers were told that information they disclosed during the surveys might be reported to child protection officers if the research team thought that ‘a child’s safety or welfare might be at risk’. All participants were offered counselling regardless of what was disclosed during the survey. Adults were offered referral to IRC services.

### Process data collected from intervention schools

During the intervention period, teachers’ attendance at each EmpaTeach session and whether they completed intervention homework was recorded by their group facilitator. Tracking sheets were delivered to headteachers and collected on a weekly basis by IRC’s programme team. IRC conducted a series of teacher classroom observations and random spot checks that, together with our survey and qualitative data, will inform a separate process evaluation that will explore the intervention’s mechanisms of action. Movement of individual teachers across schools was recorded centrally by IRC’s education team; the team was also alert to movement of groups of students, but individual student movement across schools was not tracked during the study.

### Outcomes

The primary outcome was students’ self-reports of experience of past-week physical violence from school staff at midline, measured using an adapted version of the ISPCAN Child Abuse Screening Tool–Child Institutional (ICAST-CI) [36]. Secondary outcomes were students’ self-reported experience of past-week physical violence from school staff at endline, students’ self-reported experience of past-week emotional violence from school staff at midline and endline (measured using the ICAST-CI), students’ self-reported depressive symptoms (Mood and Feelings Questionnaire [MFQ] [37] score of 12 or above) at midline and endline, and students’ self-reported past-week school attendance at midline and endline. Students were categorised as having experienced physical violence from school staff in the past week if they had experienced at least 1 out of 23 different violent behaviours in the past week. The depressive symptom scale had a good internal consistency both at midline (Cronbach’s alpha = 0.86) and endline (Cronbach’ alpha = 0.88). The exact wording of the questions used for the creation of the individual-level outcomes is provided in S1 Table.

We constructed 7 intermediate outcome variables to assess teachers’ adoption of the self-regulation and positive discipline strategies that were described in the EmpaTeach booklet, and teachers’ self-control, attitudes toward corporal punishment, and job satisfaction. Detail on the construction of these variables is included in S1 Table.

All instruments have been widely used and validated in international settings. Items were translated to Kiswahili and Kirundi, cognitively tested with a sample of 5 students and 5 teachers from the same camp population, and adapted where necessary, prior to the baseline survey. Cognitive testing participants were then excluded from the trial sampling frame.

### Statistical analysis

We performed an intention-to-treat analysis with data from our cross-sectional surveys. All analyses were done in Stata/IC 16 [29]. Analyses were done with individual student data, accounting for clustering of students within schools using generalised estimating equations (GEEs) with an exchangeable correlation structure, and robust standard errors. For binary outcomes, GEEs with a log link were fitted to estimate risk ratios (RRs), and for continuous outcomes, a Gaussian link was used. Adjusted estimates controlled for the stratification variables and also incorporated the cluster-level mean of the outcome at baseline for continuous outcomes. We also explored heterogeneity of the treatment effect by testing for an interaction with pre-specified subgroups (sex, country of origin, school level, functional difficulty, and baseline level of past-week physical violence), and conducted sensitivity analyses for missing data. Finally, in the schools that received the intervention, we explored the association between the primary outcome and school-level adherence to the intervention, defined as the proportion of EmpaTeach groups where average attendance to sessions was at least 80%. Results were considered statistically significant at the 2-sided 5% level. No formal adjustment for multiple comparisons was performed, but a limited number of pre-specified tests were conducted. Further details of the study and analysis methods are reported in the statistical analysis plan [38].

Following suggestions from reviewers, we conducted additional post hoc analyses including exploratory analyses of intermediate teacher outcomes that we hypothesized would be on the pathway to impact for the main trial outcome, analyses of the intervention’s dose and quality using monitoring data from IRC, and analyses of emotional violence that adjusted for the school-level mean of the outcome at baseline.

### Changes to protocol

Minor pragmatic changes to the trial protocol were made. We had originally intended to link data on educational outcomes for students from school records to our survey data, to explore the effects of the intervention on school achievement as a secondary outcome; however, it became clear that record linkage would be too time-consuming, and we instead analysed school attendance as a secondary outcome.

## Results

### Baseline characteristics and balance across arms

**Fig 1:**
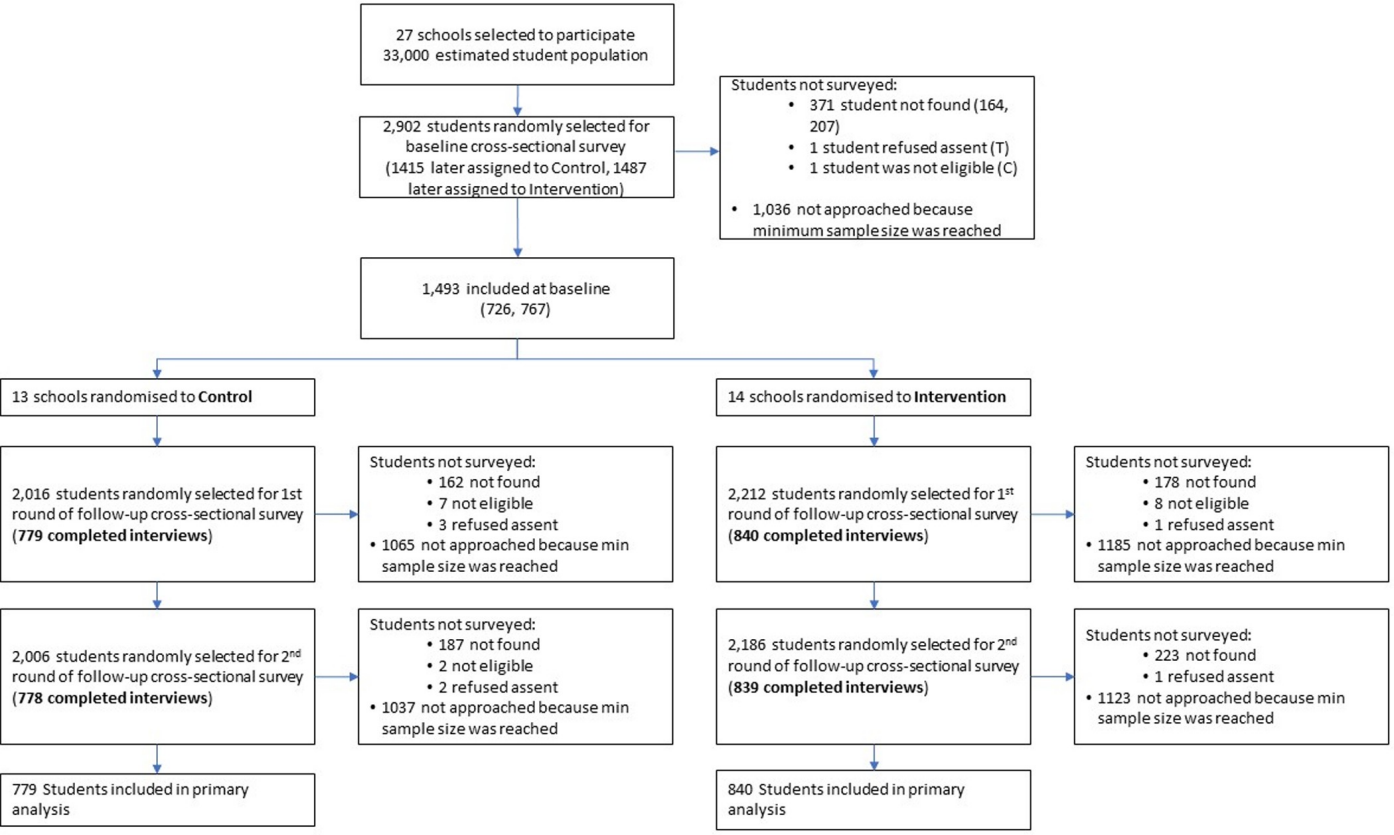
Trial flow diagram. At each round of data collection, a back-up sample of students was randomly generated following the same procedure as for the main target sample. Whenever a randomly selected respondent was not found, not available, not eligible, or refused consent, enumerators were instructed to select a replacement student from the list of back-ups. Enumerators were instructed to stop replacements when the minimum (min) sample size per school was reached. This strategy was adopted because the lists of all students enrolled were of low quality and poorly maintained; therefore, high volumes of missing students were expected. First round of follow-up cross-sectional survey = midline; second round of follow-up cross-sectional survey = endline. C, control; T, intervention.

All 27 schools in Nyarugusu participated in each of the baseline, midline, and endline surveys. At baseline 1,493 of the 1,866 (80%) randomly sampled students approached for participation took part in the survey (Fig 1). Characteristics of schools, students, and teachers were evenly distributed across arms. At baseline, students ranged from 9 to 27 years old, with a mean age of 12.9 (SD = 3.5 years), and 46.5% were female. In total, 37.8% of students were from Burundi, and 62.1% were from the Democratic Republic of the Congo; 43.8% of students reported some functional difficulty (disability). There were high levels of hunger—17.0% of students had 0 or 1 meal the previous day, and 35.5% of teachers had 0 or 1 meal the previous day. Teachers were predominately male (72.7%) and had an average age of 34.5 years (SD = 10.8 years); 84.6% were married, and most had at least 2 children. Most teachers (85.5%) had a secondary school education themselves.

Primary and secondary outcomes at baseline were similar across intervention and control arms (Table 1) for physical violence, depressive symptoms, and school attendance. In total, 54.1% of students reported past-week physical violence from a teacher, and 8.8% reported depressive symptoms. The mean number of school days attended across the 5-day Burundian school week was 4.46 (SD = 1.03), and across the 6-day Congolese school week was 4.97 (SD = 1.53). There was a slight difference for past-week emotional violence from school staff, which was reported more commonly by students in the control arm than by students in the intervention arm (20.1% and 15.6%, respectively). The characteristics of students remained balanced across the 2 intervention arms in subsequent survey rounds (S2 Table).

**Table 1:** Baseline characteristics.

Characteristic	Control		Intervention		All	
	*n* or mean	Percent or SD	*n* or mean	Percent or SD	*n* or mean	Percent or SD
**School characteristics**						
*N*	13		14		27	
Stage						
Primary	10	76.9%	11	78.6%	21	77.8%
Secondary	3	23.1%	3	21.4%	6	22.2%
Nationality						
Burundian	5	38.5%	5	35.7%	10	37.0%
Congolese	8	61.5%	9	64.3%	17	63.0%
**Student characteristics**						
*N*	726		767		1,493	
Age group (years)						
10 or below	225	31.0%	228	29.7%	453	30.3%
11–14	314	43.3%	329	42.9%	643	43.1%
15–20	157	21.6%	184	24.0%	341	22.8%
21 or above	30	4.1%	26	3.4%	56	3.8%
Sex						
Male	380	52.3%	419	54.6%	799	53.5%
Female	346	47.7%	348	45.4%	694	46.5%
Country of origin						
Burundi	283	39.0%	282	36.8%	565	37.8%
DRC	443	61.0%	484	63.1%	927	62.1%
Other	0	0.0%	1	0.1%	1	0.1%
School year						
Primary grade 1–4	223	30.7%	159	20.7%	382	25.6%
Primary grade 4–6	316	43.5%	396	51.6%	712	47.7%
Primary grade 7–9 (Burundian)	26	3.6%	46	6.0%	72	4.8%
Secondary grade 11–14 (Burundian)	53	7.3%	59	7.7%	112	7.5%
Secondary form 1–3 (Congolese)	77	10.6%	71	9.3%	148	9.9%
Secondary form 4–6 (Congolese)	31	4.3%	36	4.7%	67	4.5%
Functional difficulty	329	45.3%	325	42.4%	654	43.8%
Meals on previous day						
0 or 1	118	16.3%	136	17.7%	254	17.0%
2	502	69.1%	513	66.9%	1,015	68.0%
3 or more	106	14.6%	118	15.4%	224	15.0%
Lives without biological parents	185	25.5%	196	25.6%	381	25.5%
**Student outcomes at baseline**						
Physical violence	394	54.3%	413	53.8%	807	54.1%
Emotional violence	152	21.0%	120	15.6%	272	18.2%
Depressive symptoms	65	9.0%	67	8.7%	132	8.8%
School attendance (days)^a^^,^^b^						
Burundian schools	4.51	1.00	4.41	1.05	4.46	1.03
Congolese schools	4.93	1.56	5.00	1.51	4.97	1.53
**Teacher characteristics**						
*N*	235		253		488	
Age (years)	34.2	10.8	34.7	10.8	34.5	10.8
Sex						
Male	169	71.9%	186	73.5%	355	72.7%
Female	66	28.1%	67	26.5%	133	27.3%
Country of origin						
Burundi	92	60.8%	92	63.2%	184	62.1%
DRC	143	39.1%	160	36.4%	303	37.7%
Other	0	0.0%	1	0.4%	1	0.2%
Religion^a^						
Pentecostal	57	24.6%	63	25.3%	120	24.9%
Roman Catholic	52	22.4%	56	22.5%	108	22.5%
Anglican	25	10.8%	16	6.4%	41	8.5%
Other	98	42.2%	114	45.8%	212	44.1%
Marital status						
Single	33	14.0%	32	12.6%	65	13.3%
Married	197	85.3%	216	85.4%	413	84.6%
Separated/widowed	5	2.1%	5	2.0%	10	2.0%
Number of children^a^						
0	22	9.8%	23	9.7%	45	9.7%
1–2	80	35.7%	87	36.6%	167	36.1%
3–5	70	31.3%	70	29.4%	140	30.3%
6 or more	52	23.2%	58	24.4%	110	23.8%
Highest qualification						
University degree	29	12.3%	38	15.0%	67	13.7%
Secondary school	206	87.7%	211	83.4%	417	85.5%
Below secondary	0	0.0%	4	1.6%	4	0.8%
Meals on previous day						
0 or 1	88	37.4%	85	33.6%	173	35.5%
2	134	57.0%	143	56.2%	277	56.8%
3 or more	13	5.5%	25	9.9%	38	7.8%

DRC, Democratic Republic of the Congo; SD, standard deviation.

^a^Missing data: student’s school attendance, *n =* 11; teacher’s religion, *n =* 7; teacher’s number of children, *n =* 26.

^b^Number of days attended schools in the last week. Typically there are 5 school days per week in Burundian schools and 6 days per week in Congolese schools.

### Intervention delivery and adherence

Across the 14 intervention schools, 600 teachers formed 77 EmpaTeach groups (Table 2). Around half of the teachers (52.4%) attended between 10 and 12 of the total 12 sessions, and a median of 8 teachers (range 3 to 15) were assigned to each group. More than 90% of groups had an average attendance of more than 50% (40.3% of groups had an average attendance between 50% and 80%, and 53.2% of groups had an average attendance of 80% or more). At the school level, 35.7% of schools had ‘high’ levels of intervention delivery, with 70% or more of their EmpaTeach groups reporting 80% or more attendance. In most cases, groups included teachers who were from the same school, but some of those who received the compressed intervention were in groups with teachers from different schools.

**Table 2:** Intervention delivery and fidelity.

Measure	*n/N* or median	Percent or range
**Number of sessions attended by teachers** ^**a**^		
0–5 sessions	99/597	16.6%
6–9 sessions	185/597	31.0%
10–12 sessions	313/597	52.4%
**Average group size**	8	3 to 15
**Average attendance rate at the group level**		
<50% of group members attended	5/77	6.5%
50%–80% of group members attended	31/77	40.3%
≥80% of group members attended	41/77	53.2%
**Number of EmpaTeach groups per school**	5	3 to 11
**EmpaTeach version**		
Schools that received EmpaTeach original only	8	57.1%
Schools that received EmpaTeach original or compressed^b^	6	42.9%
**School level proportion of groups with ≥80% attendance**		
<30%	4	28.6%
30–69%	5	35.7%
≥70%	5	35.7%

^a^A total of 600 teachers took part in the intervention, but attendance data were missing for 3 teachers; analyses were conducted on 597 teachers with attendance data available.

^b^In those 6 schools, between 1 out of 7 groups (14%) and 3 out of 10 groups (30%) received the compressed version.

Levels of teacher movement during the intervention were minimal. Among teachers who were originally in control schools, 4 moved to an intervention school and joined an existing group. Among the teachers who were originally in an intervention school, 11 moved across treatment schools, 5 moved to control schools, and 10 retired or were promoted to management roles.

We had 1 major incident that affected the delivery of the intervention. Owing to a camp-wide policy change, approximately 20% of teachers in the camp were made redundant (laid off) from their jobs at a single time point in January 2019, during the intervention delivery period. The vast majority were then re-hired in the same role in the same school within a period of 2 weeks; however, some were not. This disrupted the functioning of the EmpaTeach groups over a period of approximately 4 weeks. IRC’s programme delivery team created a compressed version of the programme for teachers affected by the layoff, in which the same intervention content was delivered over a shorter time period (6 weeks instead of 10). Overall, 14.7% of intervention teachers received the compressed version, and 85.3% of teachers received the original version of EmpaTeach over 10 weeks.

S3 Table provides additional descriptive indicators of intervention dose and quality. Despite reasonable attendance rates and punctual use of materials and booklets by the majority of participating teachers, data from IRC’s spot checks conducted during the intervention sessions showed that there were large gaps in homework completion and timeliness.

### Main results

Results from all primary and secondary outcome analyses are reported in Table 3. At midline, 412 of 777 (53.0%) students in control schools and 408 of 839 (48.6%) students in intervention schools reported past-week physical violence from teachers, corresponding to an adjusted RR of 0.91 (95% CI 0.80 to 1.02), which was not statistically significant (*p* = 0.106). There were no statistically significant differences in any of the secondary outcomes tested at midline or endline, with the exception of school attendance at endline, which reached borderline statistical significance. At endline, students in intervention schools appeared to attend slightly less school than students in control schools, with a mean difference of −0.19 days (95% CI −0.39 to 0.00, *p* = 0.049). Also, although not statistically significant, there was a slightly higher prevalence of physical violence in the intervention schools at endline (RR = 1.11, 95% CI 0.99 to 1.25, *p* = 0.077).

**Table 3:** Main trial results.

Outcome	Control		Intervention		Comparison				
					Unadjusted		Adjusted^a^		
	Percent or mean	*n/N* or SD	Percent or mean	*n/N* or SD	RR difference	95% CI	RR difference	95% CI	*p-*value
**Primary outcome**									
Physical violence at midline	53.0%	412/777	48.6%	408/839	0.92	0.72 to 1.17	0.91	0.80 to 1.02	0.106
**Secondary outcomes—midline**									
Emotional violence	20.7%	161/777	18.4%	154/839	0.89	0.67 to 1.17	0.88	0.70 to 1.11	0.280
Depressive symptoms	12.2%	95/778	12.5%	105/840	1.02	0.76 to 1.38	1.02	0.79 to 1.33	0.862
School attendance									
Burundian student	4.29	1.02	4.25	1.27	−0.04	−0.51 to 0.42	−0.06	−0.18 to 0.05	0.275
Congolese student	5.15	1.23	5.06	1.21	−0.09	−0.27 to 0.09			
**Secondary outcomes—endline**									
Physical violence	47.8%	372/778	53.9%	452/839	1.13	0.90 to 1.42	1.11	0.99 to 1.25	0.077
Emotional violence	18.5%	144/778	15.4%	129/839	0.83	0.62 to 1.11	0.83	0.64 to 1.08	0.172
Depressive symptoms	10.3%	80/778	11.0%	92/839	1.07	0.74 to 1.53	1.06	0.76 to 1.48	0.738
School attendance									
Burundian student	4.58	0.77	4.55	0.81	−0.03	−0.15 to 0.10	−0.19	−0.39 to 0.00	0.049
Congolese student	5.28	1.08	4.98	1.22	−0.30	−0.61 to 0.01			

CI, confidence interval; RR, risk ratio. Control group includes 13 schools, 779 students surveyed at midline, and 778 students surveyed at endline. Intervention group includes 14 schools, 840 students surveyed at midline, and 839 students surveyed at endline. Missing data for midline survey: physical violence, *n =* 3; emotional violence, *n =* 3; depression, *n =* 1; attendance, *n =* 5. Missing data for endline survey: attendance, *n =* 1.

^a^Adjusted for randomisation strata (school nationality and level); analyses of the school attendance outcome are additionally adjusted for baseline school-level mean attendance. The mixed-effects logistic regression estimate of the intra-cluster correlation coefficient (ICC) for the primary outcome was 0.13. Adjusting the emotional violence analyses by baseline school-level mean of the outcome produced the following estimates: at midline, adjusted RR = 0.99 (95% CI 0.83 to 1.19, *p* = 0.947), and at endline, adjusted RR = 0.80 (95% CI 0.62 to 1.03, *p* = 0.083).

### Exploratory adherence analyses

For the 14 schools in the intervention arm, we explored whether schools with a higher level of EmpaTeach delivery reported less student experience of physical violence. There was no statistically significant association with the school proportion of high-attendance groups or with the school proportion of groups receiving the compressed version of EmpaTeach (Table 4).

**Table 4:** Adherence analyses.

Measure	Number of schools	Physical violence at midline			*p-*Value^b^
		*n/N*	Percent^a^	95% CI^a^	
**Percent of groups with ≥80% attendance**					0.510
<30%	4	129/240	53.7%	45.3 to 63.8	
30%–69%	5	136/299	45.5%	38.5 to 53.7	
≥70%	5	143/300	47.7%	31.7 to 71.7	
**EmpaTeach version**					0.396
Original only	8	253/479	52.8%	46.3 to 60.2	
Original or compressed	6	155/360	43.1%	30.6 to 60.5	

CI, confidence interval.

^a^Percentage and confidence interval based on generalised estimating equations.

^b^*p-*Value testing for a linear association with the school-level proportion of groups with attendance at or above 80% or receiving the original EmpaTeach version.

### Subgroup analyses

We planned a priori to explore whether the effects of the intervention differed in male and female students, in students who reported a functional difficulty (disability), by school nationality, by school stage, and by school baseline level of physical violence. No subgroup effects were statistically significant (all interaction *p-*values ≥ 0.46) (S4 Table).

### Exploratory intermediate outcome analyses

We conducted exploratory post hoc analyses to investigate whether the intervention affected intermediate outcomes for teachers (Table 5). Generally, teachers in intervention schools used more positive discipline strategies than teachers in control schools both at midline and endline; however, the size of the effect was very small. Teachers in schools that received EmpaTeach also reported better self-regulation at endline compared to control teachers, and attitudes less supportive of physical violence at both midline and endline, but again, the differences between intervention and control groups were very small. No effect was observed on job satisfaction.

**Table 5:** Exploratory analyses of intermediate outcomes for teachers.

Outcome	Midline				Endline			
	Mean (SD)		Comparison		Mean (SD)		Comparison	
	Control	Intervention	Marginal effect (95% CI)^a^	*p-*Value	Control	Intervention	Marginal effect (95% CI)^a^	*p-*Value
Number of positive discipline acts to manage stress in class (range 0–5)	0.84 (0.81)	1.22 (0.86)	0.36 (0.11 to 0.62)	0.01	1.08 (0.89)	1.33 (0.94)	0.25 (0.11 to 0.38)	0.00
Number of positive discipline acts to manage students disturbing in class (range 0–8)	1.21 (1.04)	1.55 (1.01)	0.34 (0.06 to 0.62)	0.02	1.50 (1.15)	1.65 (1.23)	0.14 (−0.13 to 0.42)	0.31
Number of positive discipline acts to manage students arriving late to class (range 0–2)	0.11 (0.34)	0.18 (0.43)	0.07 (−0.01 to 0.15)	0.09	0.05 (0.23)	0.10 (0.32)	0.05 (0.01 to 0.09)	0.02
Number of positive discipline acts to praise students (range 0–10)	1.98 (1.06)	2.11 (0.99)	0.15 (−0.11 to 0.40)	0.26	1.90 (0.92)	2.05 (1.04)	0.16 (−0.10 to 0.41)	0.22
Self-regulation score (1–5)	3.86 (0.42)	3.90 (0.44)	0.05 (−0.03 to 0.12)	0.22	3.96 (0.42)	4.03 (0.43)	0.07 (0.01 to 0.13)	0.02
Job satisfaction (1–4)	2.67 (0.71)	2.60 (0.74)	−0.08 (−0.25 to 0.08)	0.30	2.52 (0.69)	2.45 (0.70)	−0.07 (−0.21 to 0.07)	0.31
Attitudes towards corporal punishment (1–4)	2.90 (0.57)	3.03 (0.44)	0.13 (0.03 to 0.24)	0.01	2.82 (0.56)	2.94 (0.53)	0.12 (0.02 to 0.23)	0.02

CI, confidence interval; SD, standard deviation. Control group includes 13 schools, 245 teachers surveyed at midline, and 314 teachers surveyed at endline. Intervention group includes 14 schools, 265 teachers surveyed at midline, and 354 teachers surveyed at endline. Missing data for endline job satisfaction: *n =* 1.

^a^Adjusted for randomisation strata (school nationality and level). Marginal effect and confidence interval based on generalised estimating equations.

### Sensitivity analyses

We performed sensitivity analyses to explore the effects of missing data on our primary outcome—assuming that all students with missing data for that outcome were students who had experienced physical violence—and the effects of modelling the MFQ score as a continuous variable. Missing data and MFQ modelling had little effect on results (S5 Table).

### Adverse effects

No adverse effects of the intervention itself were detected. Across our 3 cross-sectional surveys, 765 (398 control and 367 intervention) of 4,729 students were referred to child protective services because of violence they disclosed during the survey data collection.

## Discussion

### Summary of main findings

We did not find evidence that the EmpaTeach intervention reduces physical violence from teachers, as reported by students in Nyarugusu Refugee Camp, Tanzania. There was no suggestion that schools that had higher levels of intervention delivery had lower levels of violence, and we also did not find any consistent effects of the intervention on emotional violence, student’s depressive symptoms, or school attendance. The intervention appeared to have positively influenced intermediate outcomes for teachers such as use of positive discipline, self-regulation, and attitudes towards violence; however, the effect sizes were generally very small and not likely to be practically meaningful.

### Contribution to the literature

To our knowledge, EmpaTeach represents 1 of only 3 interventions aimed at reducing violence from teachers to students to be formally tested in a rigorous cluster-randomised controlled trial, and the first trial of any programme aimed at reducing violence against children in schools in a humanitarian setting.

A number of factors related to the camp context, the intervention design, and how the intervention was delivered may help explain our results. Conditions in Nyarugusu differ substantially from non-refugee settings, which may have affected teachers’ ability to engage with the intervention. Refugees are not allowed to engage in formal employment and are unable to leave the camp area unless issued with special permits [39], and thus are compensated with small incentives rather than a salary. Levels of hunger in the camp are high, with more than a third of teachers eating 1 or 0 meals the previous day. Overcrowding is also a widespread problem in already severely resource-constrained schools [5]. The vast majority of teachers also engage in other income-generating activities beyond their teaching role at the school (at baseline teachers reported working on average 11 hours per week in other paid jobs). Teachers are from the camp population, and most are not professionally qualified. All teachers in intervention schools were asked to participate in the intervention sessions, regardless of their interest in the content of the programme or their ability to engage in it. Although levels of attendance at groups were reasonable, according to some indicators there were limited levels of participation and commitment among groups, which may have hindered the overall success of the intervention. Completion of homework practice was essential to promote the adoption of positive behaviours, and group exchange during weekly sessions was thought to be important to generate social support and reinforce positive norms. Patchy attendance and engagement may have compromised the effectiveness of both mechanisms. Teachers’ levels of commitment to their schools and professional development were also likely negatively affected by the mass redundancy and re-hiring of teachers during intervention implementation. Although some schools were located close to other schools, the risk of intervention contamination of control schools was considered to be low given the nature of the intervention, which relied on facilitated participation and group interactions.

The content of EmpaTeach has important similarities and differences to the rigorously trialled and effective Irie Classroom Toolbox [21] and Good School Toolkit [22,40] interventions to reduce teacher violence. Similar to these interventions, and as recommended by WHO’s school-based violence prevention guidance [41], EmpaTeach contains information on alternative discipline techniques and creates social support.

In contrast to the Good School Toolkit, EmpaTeach does not involve school management structures and does not focus on normative beliefs around violence. It takes a similar strategy to the Irie Classroom Toolbox, based on promoting positive classroom management practices and teacher well-being. Similar to the Irie Classroom Toolbox, EmpaTeach purposefully avoids directly discouraging the use of violence in all intervention materials; instead, this topic is addressed indirectly through stories and reflection exercises included in the teacher booklet. Our exploratory analyses of teachers’ use of positive discipline showed that teachers in EmpaTeach schools adopted some recommended alternative approaches. However, the lack of explicit discouragement of using corporal punishment may have resulted in an expansion of teachers’ disciplinary methods rather than in the replacement of violent discipline. In other words, it is possible that the positive disciplinary techniques adopted by teachers were used alongside traditional corporal punishment methods, rather than as replacement for violence.

Both the Good Schools Toolkit and the Irie Classroom Toolbox also had longer and more intensive periods of delivery than EmpaTeach, which was much shorter and involved less facilitation and mentoring. EmpaTeach may have benefitted from more time to allow the small observed changes in intermediate outcomes to translate into changes in levels of violence. The light-touch, teacher-led approach of EmpaTeach was implemented by peer teachers who received 3 days of training, which may not have been sufficient on its own to prepare facilitators to support peers through a complex process of behaviour change. Both the Good School Toolkit and the Irie Classroom Toolbox begin with intensive training, but in both cases the initial training is provided by external facilitators, and perhaps crucially, follow-up support is provided for 18 and 8 months for the Good School Toolkit and the Irie Classroom Toolbox, respectively. The lack of external expert facilitation and continued support in EmpaTeach may have also contributed to limiting facilitators’ ability to support and mentor teachers throughout the intervention duration.

Evidence from school-based interventions in other areas of public health [41,42] also suggests that commitment from school administration and the creation of school-based systems that promote change at the institutional level are key enabling factors. In the case of EmpaTeach, school headteachers and discipline teachers received a 1-day training about the intervention. Teacher–parent associations and other school administrators were informed about the intervention, but were not actively engaged in the programme; this was done to enhance the cost-efficiency of the intervention. The lack of external accountability and limited school-level support to reinforce and normalise the newly acquired positive disciplinary methods may also have limited teachers’ ability to adopt new skills and mindsets.

### Strengths and limitations

Our study has both strengths and limitations. We randomly allocated schools to trial arms, no schools left the study, and student and teacher response rates were high. We had low levels of missing data. Although we were able to include a complete sample of all schools in Nyarugusu Refugee Camp, our statistical power was limited by the relatively small number of schools. We cannot rule out a small beneficial effect of the intervention on the primary outcome of physical violence at midline, or a small harmful effect of the intervention on physical violence or school attendance at endline. However, the direction and coherence of the results for our secondary outcomes, coupled with the small size of the observed changes in the primary, secondary, and intermediate outcomes, suggest to us that EmpaTeach did not have an effect in this study.

We did have 1 major incident that affected intervention delivery, which is perhaps expected, given the challenges inherent in working in humanitarian settings. Our adherence analyses did not detect any differential effects of the compressed versus original format intervention, but we would have been underpowered to detect a difference, as it was necessary to conduct analyses at the school level. We used internationally accepted, standardised questions to ask students about their experiences of violence; however, similar to other studies on violence, we had to rely on self-reports as no better measure exists. Test–retest reliability of the ICAST-CI is not well established, similar to other commonly used violence measures. Similar to other violence prevention trials, we did not investigate test–retest reliability in our sample, which is a limitation. Lack of reliable reporting of violence exposure could have biased estimates towards or away from a null effect, which may have further limited our ability to detect differences between the control and intervention groups in this study. Although the intervention directly engages teachers, we used students’ self-reports of violence as the primary outcome because teachers may have been motivated to under-report their use of violence due to exposure to the intervention rather than their own behaviour change [43]. Our pre-specified trial analyses of the main outcome and binary secondary outcomes were unadjusted for baseline levels; however, baseline differences between schools were very small. Similar to other studies of behavioural interventions, we were unable to mask intervention recipients and interviewers to allocation. A further limitation is that no census of children attending school in the camp exists; hence, it was not possible to assess how representative of the overall camp school population our sample was, although it was based on a stratified random sample with a relatively high response rate. Our results should not be interpreted as generalisable to all humanitarian settings, as Nyarugusu is an older and more stable and established camp relative to some other refugee settings.

### Implications for future research

Evidence on effective solutions to prevent and reduce teacher violence in schools continues to be limited, and it remains unclear to what extent and under what circumstances teacher violence against students across various contexts is performed because it is a normative, entrenched behaviour; when it is driven by an impulsive loss of control; and when it is a combination of both factors. Quantitative analyses show that violent behaviour clusters within individual teachers [44], suggesting that self-regulation or other individual traits may play an important role. However, qualitative work also shows the widespread and normative nature of corporal punishment in schools, suggesting that the majority of violent acts may not be immediately caused by loss of teacher control [45]. Open questions remain about how best to address both of these pathways in interventions to reduce teacher violence. Further exploration of the relative importance of these motives (loss of self-control versus normative attitudes and behaviours) in different contexts is warranted. We also note that a recently published evaluation of the Right to Play intervention to empower children by teaching communication and conflict resolution skills in Pakistan showed a reduction in children’s experience of corporal punishment from teachers, even though the intervention did not directly engage with teachers [46]. Future intervention work to reduce teacher violence may also usefully explore alternative pathways to violence reduction.

## Conclusion

Despite a total ban on use of corporal punishment in Nyarugusu schools, levels of physical violence from teachers remain high, and similar to surrounding countries [11–13,47]. We did not find evidence that the EmpaTeach intervention was effective in reducing teachers’ use of physical violence against students in Nyarugusu Refugee Camp. Effective interventions are necessary to reduce this extremely common form of child maltreatment, and further trials of a range of different intervention models are urgently needed. Testing should explore how far implementation time can be reduced to come up with a minimum effective package that can be implemented in a range of settings, including in refugee settings, and should investigate the role of different intervention elements in producing change.

## Supporting information

S1 CONSORT Checklist (DOCX)

S1 Fig EmpaTeach theory of change. The figure illustrates how EmpaTeach activities were hypothesized to lead to improvements in teacher well-being and reduce levels of violence against students.
(TIF)

S2 Fig Subgroup analyses. The faded horizontal lines represent the level of uncertainty around each point estimate. They do not correspond to a particular confidence level (e.g., 95%) to avoid focusing on whether they cross the ‘no difference’ axis, which would be misleading within subgroups [48].
(TIF)

S1 Table Description of study outcomes. (DOCX)

S2 Table Student characteristics at midline and endline follow-up. (DOCX)

S3 Table Indicators of intervention dose and quality. (DOCX)

S4 Table Subgroup analyses. (DOCX)

S5 Table Sensitivity analyses. (DOCX)

S1 TIDieR Checklist (DOCX)

## Abbreviations

ICAST-CI: ISPCAN Child Abuse Screening Tool–Child Institutional
IRC: International Rescue Committee
MFQ: Mood and Feelings Questionnaire
PVACS: Preventing Violence Against Children in Schools
RR: risk ratio
