# Supplementary figures and images for: The EmpaTeach intervention for reducing physical violence from teachers to students in Nyarugusu Refugee Camp: A cluster-randomised controlled trial
Source: PLoS Med. 2021 Oct 4;18(10):e1003808. doi: 10.1371/journal.pmed.1003808 (PMC8489723; doi:10.1371/journal.pmed.1003808)

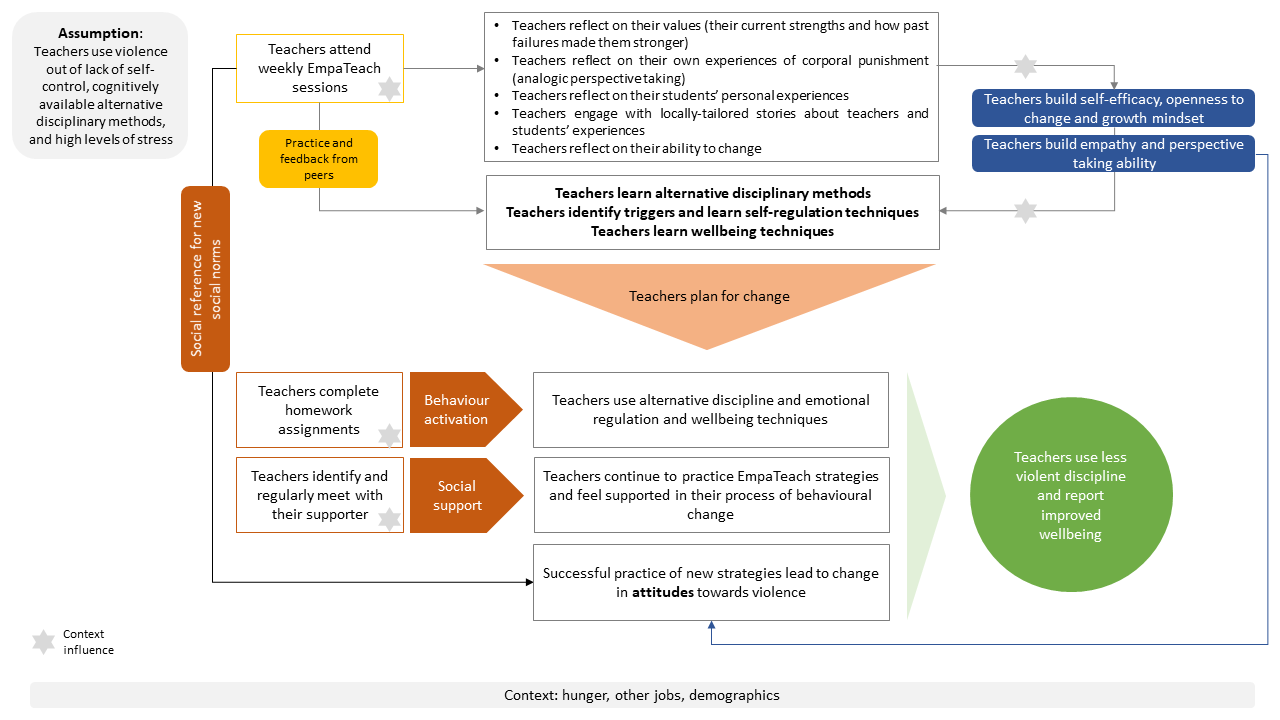

Supplement: S1 Fig — The figure illustrates how EmpaTeach activities were hypothesized to lead to improvements in teacher well-being and reduce levels of violence against students. (TIF) [file pmed.1003808.s002.tif]

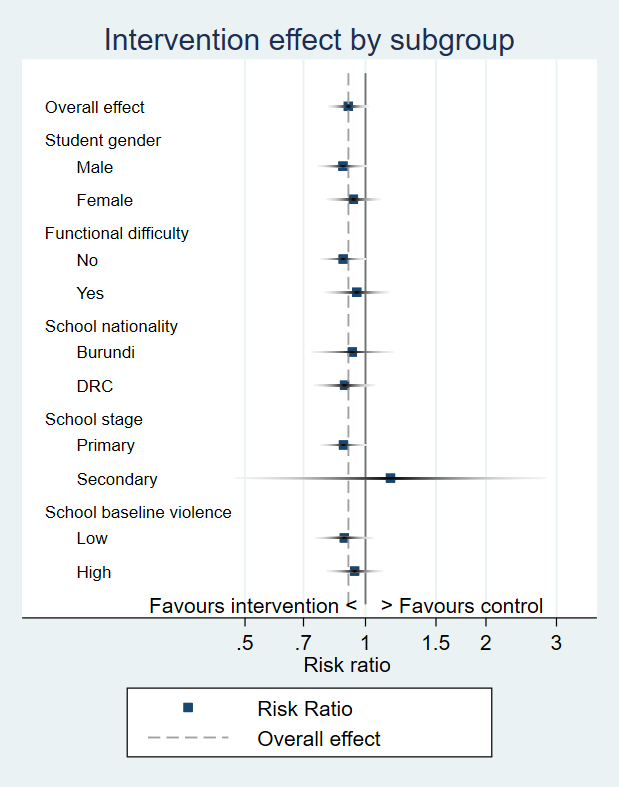

Supplement: S2 Fig — The faded horizontal lines represent the level of uncertainty around each point estimate. They do not correspond to a particular confidence level (e.g., 95%) to avoid focusing on whether they cross the ‘no difference’ axis, which would be misleading within subgroups [48]. (TIF) [file pmed.1003808.s003.tif]
