# Supplementary material for: The EmpaTeach intervention for reducing physical violence from teachers to students in Nyarugusu Refugee Camp: A cluster-randomised controlled trial
Source: PLoS Med. 2021 Oct 4;18(10):e1003808. doi: 10.1371/journal.pmed.1003808 (PMC8489723; doi:10.1371/journal.pmed.1003808)
Supplement: S1 Table — (DOCX) [file pmed.1003808.s004.docx]

S1 Table. Description of study outcomes

| Variable | Items | Coding |
| --- | --- | --- |
| Primary outcome | | |
| Physical violence from school staff in past week(1) | Hurt you or caused pain to you? Slapped you with a hand on your face or head as punishment? Slapped you with a hand on your arm or hand? Twisted your ear as punishment? Twisted your arm as punishment? Pulled your hair as punishment? Hit you by throwing an object at you? Hit you with a closed fist? Hit you with a stick? Hit you with a cane? Kicked you with a foot? Knocked you on the head as punishment? Hit your fingers or hands with an object as punishment? Crushed your fingers or hands as punishment? Made you stand/kneel in a way that hurts to punish you? Made you stay outside for example in the heat or rain to punish you? Burnt you as punishment? Taken your food away from you as punishment? Forced you to do something that was dangerous? Choked you? Tied you up with a rope or belt at school? Tried to cut you purposefully with a sharp object? Severely beat you up? | Coded 1 if answered yes about past week experience to any of the items; coded 0 if answered no to all items. |
| Secondary outcomes | | |
| Emotional violence from school staff in past week(1) | Cursed or called you bad names? Shouted or yelled at you? Insulted you or said something mean to you? Humiliated or embarrassed you? Talked about your skin colour/gender/religion/tribe or health problems you have in a hurtful way? Kept you away from other children to make you feel bad or lonely? Humiliated you because you were unable to buy things? Stole or broke or ruined your belongings? Threatened you with bad marks that you didn’t deserve? | Coded 1 if answered yes about past week experience to any of the items; coded 0 if answered no to all items. |
| Depression symptoms (Mood and Feelings Questionnaire)(2) | In the past two weeks, you felt very sad, In the past two weeks, you didn’t enjoy anything at all,  In the past two weeks, you could not do your normal activities because you felt so sad,  In the past two weeks, you could not relax your mind, In the past two weeks, you felt worthless,  In the past two weeks, you cried a lot, In the past two weeks, you found it hard to pay attention, In the past two weeks, you hated yourself, In the past two weeks, you were a bad person, In the past two weeks, you felt sad because you were alone with your thoughts, In the past two weeks, you thought nobody really loved you, In the past two weeks, you thought you could never be as good as other kids, In the past two weeks, you did everything wrong. | Each item was scored according to the scaled: 0=not true, 1=sometimes true, 2=true.  Students screened positive for depression symptoms if they reported a total score of 12 or higher. |
| School attendance in past week | In the past week (7 days) on how many days did you attend school? | Count of days reported |
| Intermediate exploratory outcomes | | |
| Teacher adoption of positive discipline strategies | Each variable was constructed as a continuous measure comprised of the number of positive practices teachers reported using in various situations. | Count of positive practices |
| Teacher self-regulation (Brief Self Control Scale)(3) | I am good at resisting temptation.  (R) I have a hard time breaking bad habits.  (R) I am lazy.  (R) I say inappropriate things.  (R) I do certain things that are bad for me, if they are fun.  I refuse things that are bad for me.  (R) I wish I had more self-discipline. (removed at endline)  People would say that I have iron self-discipline. (removed at endline)  (R) Pleasure and fun sometimes keep me from getting work done. (removed at endline)  (R) I have trouble concentrating. (removed at endline)  I am able to work efficiently towards long-term goals.  (R) Sometimes I can’t stop myself from doing something, even if I know it is wrong. | Mean score based on a 5 points Likert scale.  (Cronbach’s alpha = 0.59) |
| Teacher attitudes towards corporal punishment (Attitudes on “Child Beating)(4) | It is acceptable to hit a student:  If the student is interrupting class  if the student talks back to the teacher,  If the student is absent from school,  If the student insults the teacher  If the student is not taking notes during a lesson,  If the student fights with other students | Mean score based on a 4 points Likert scale.  (Cronbach’s alpha = 0.89) |
| Teacher wellbeing in school - job satisfaction(5) | How would you say that you enjoy your job?, Do you feel valued as an employee?, Do you take pride in your work?, Do you feel that your employers care about your well-being?, Do you feel adequately rewarded financially for what you do?. | Mean score based on a 4 points Likert scale.  (Cronbach’s alpha = 0.68) |

1. Zolotor AJ, Runyan DK, Dunne MP, Jain D, Peturs HR, Ramirez C, et al. ISPCAN Child Abuse Screening Tool Children’s Version (ICAST-C): Instrument development and multi-national pilot testing. Child Abuse Negl 2009;33(11):833–41.

2. Angold A, Costello EJ, SC. M. Development of a short questionnaire for use in epidemiological studies of depression in children and adolescentsx. International Journal of Methods in Psychiatric Research (1995), 5:237-249.

3. Tangney JP, Baumeister RF, Boone AL. High self-control predicts good adjustment, less pathology, better grades, and interpersonal success. . J Pers 2004;72, 271–324. doi: 10.1111/j.0022-3506.2004.00263.x.

4. Ruis-Caseres M. Child Protection Knowledge, Attitudes and Practices in Central and Western Liberia.” Save the Children. 2011.

5. Devries K.M., et al. The Good School Toolkit for reducing physical violence from school staff to primary school students: A cluster-randomised controlled trial in Uganda. . Lancet Global Health, 2015 3(7): p E378-86.
