# Supplementary material for: The EmpaTeach intervention for reducing physical violence from teachers to students in Nyarugusu Refugee Camp: A cluster-randomised controlled trial
Source: PLoS Med. 2021 Oct 4;18(10):e1003808. doi: 10.1371/journal.pmed.1003808 (PMC8489723; doi:10.1371/journal.pmed.1003808)
Supplement: S2 Table — (DOCX) [file pmed.1003808.s005.docx]

S2 Table. Students characteristics at midline and endline follow-up

|  | **Control** | | **Intervention** | | **All** | |
| --- | --- | --- | --- | --- | --- | --- |
|  | n | (%) | n | (%) | n | (%) |
| **Students characteristics** |  |  |  |  |  |  |
| **Midline** |  |  |  |  |  |  |
| **N** | **779** |  | **840** |  | **1,619** |  |
| Age group |  |  |  |  |  |  |
| *10 or below* | 193 | (24.8%) | 190 | (22.6%) | 383 | (23.7%) |
| *11- 14* | 336 | (43.1%) | 363 | (43.2%) | 699 | (43.2%) |
| *15 -20* | 236 | (30.3%) | 277 | (33.0%) | 513 | (31.7%) |
| *21 or above* | 14 | (1.8%) | 10 | (1.2%) | 24 | (1.5%) |
| Sex |  |  |  |  |  |  |
| *Male* | 389 | (49.9%) | 438 | (52.1%) | 827 | (51.1%) |
| *Female* | 390 | (50.1%) | 402 | (47.9%) | 792 | (48.9%) |
| Country of origin |  |  |  |  |  |  |
| *Burundi* | 300 | (38.5%) | 309 | (36.8%) | 609 | (37.6%) |
| *DRC* | 478 | (61.4%) | 531 | (63.2%) | 1,009 | (62.3%) |
| *Other* | 1 | (0.1%) | 0 | (0%) | 1 | (0.1%) |
| School year |  |  |  |  |  |  |
| *Grade 1-3* | 245 | (31.5%) | 228 | (27.1%) | 473 | (29.2%) |
| *Grade 4-6* | 344 | (44.2%) | 390 | (46.4%) | 734 | (45.3%) |
| *Grade 7-14 (Burundian)* | 69 | (8.9%) | 102 | (12.1%) | 171 | (10.6%) |
| *Form 1-6 (Congolese)* | 121 | (15.5%) | 120 | (14.3%) | 241 | (14.9%) |
| Functional difficulty |  |  |  |  |  |  |
| *Yes* | 287 | (36.8%) | 301 | (35.8%) | 588 | (36.3%) |
| Meals on previous day |  |  |  |  |  |  |
| *0-1* | 152 | (19.5%) | 142 | (16.9%) | 294 | (18.2%) |
| *2* | 487 | (62.5%) | 535 | (63.7%) | 1,022 | (63.1%) |
| *3 or more* | 140 | (18.0%) | 163 | (19.4%) | 303 | (18.7%) |
| Lives without biological parents |  |  |  |  |  |  |
| *Yes* | 167 | (21.4%) | 146 | (17.4%) | 313 | (19.3%) |
| **Endline** |  |  |  |  |  |  |
| **N** | **778** |  | **839** |  | **1,617** |  |
| Age group |  |  |  |  |  |  |
| *10 or below* | 153 | (19.7%) | 184 | (21.9%) | 337 | (20.8%) |
| *11- 14* | 379 | (48.7%) | 376 | (44.8%) | 755 | (46.7%) |
| *15 -20* | 226 | (29.0%) | 262 | (31.2%) | 488 | (30.2%) |
| *21 or above* | 20 | (2.6%) | 17 | (2.0%) | 37 | (2.3%) |
| Sex |  |  |  |  |  |  |
| *Male* | 365 | (46.9%) | 413 | (49.2%) | 778 | (48.1%) |
| *Female* | 413 | (53.1%) | 426 | (50.8%) | 839 | (51.9%) |
| Country of origin |  |  |  |  |  |  |
| *Burundi* | 307 | (39.5%) | 304 | (36.2%) | 611 | (37.8%) |
| *DRC* | 471 | (60.5%) | 535 | (63.8%) | 1,006 | (62.2%) |
| *Other* | 0 | (0%) | 0 | (0%) | 0 | (0%) |
| School year |  |  |  |  |  |  |
| *Grade 1-3* | 190 | (24.4%) | 193 | (23.0%) | 383 | (23.7%) |
| *Grade 4-6* | 391 | (50.3%) | 396 | (47.2%) | 787 | (48.7%) |
| *Grade 7-14 (Burundian)* | 77 | (9.9%) | 129 | (15.4%) | 206 | (12.7%) |
| *Form 1-6 (Congolese)* | 120 | (15.4%) | 121 | (14.4%) | 241 | (14.9%) |
| Functional difficulty |  |  |  |  |  |  |
| *Yes* | 210 | (27.0%) | 232 | (27.7%) | 442 | (27.3%) |
| Meals on previous day |  |  |  |  |  |  |
| *0-1* | 133 | (17.1%) | 140 | (16.7%) | 273 | (16.9%) |
| *2* | 523 | (67.2%) | 575 | (68.5%) | 1,098 | (67.9%) |
| *3 or more* | 122 | (15.7%) | 124 | (14.8%) | 246 | (15.2%) |
| Lives without biological parents |  |  |  |  |  |  |
| *Yes* | 219 | (28.1%) | 217 | (25.9%) | 436 | (27.0%) |

Notes: DRC= Democratic Republic of the Congo; No missing data
