# Supplementary material for: The EmpaTeach intervention for reducing physical violence from teachers to students in Nyarugusu Refugee Camp: A cluster-randomised controlled trial
Source: PLoS Med. 2021 Oct 4;18(10):e1003808. doi: 10.1371/journal.pmed.1003808 (PMC8489723; doi:10.1371/journal.pmed.1003808)
Supplement: S3 Table — (DOCX) [file pmed.1003808.s006.docx]

S3 Table. Indicators of intervention dose and quality

|  | **mean**  **n/N** | **Range**  **(%)** |
| --- | --- | --- |
| **Random spot-checks data** |  |  |
| *Session planning and coordination* |  |  |
| Session started on time | 22/59 | 37% |
| Coordinator brought materials needed | 48/59 | 81% |
| All teachers arrived on time | 25/59 | 42% |
| All teachers brought their booklet | 48/59 | 81% |
| *Engagement with intervention content* |  |  |
| Teachers completed homework |  |  |
| All teachers | 33/58 | 56% |
| Some teachers | 15/59 | 25% |
| None of the teachers | 11/59 | 19% |
| Teachers completed all activities | 49/59 | 83% |
| Teachers had trouble completing activities | 6/59 | 10% |
| Teachers had trouble understanding instructions | 3/59 | 5% |
| *Group dynamics* |  |  |
| Teachers discussed hard punishment | 14/59 | 24% |
| Teachers disagreed with booklet materials | 2/59 | 3% |
| All teachers participated in discussions | 50/59 | 85% |
| All teachers participated in roleplay | 19/27 | 70% |

Notes: This table uses data from 59 spot-checks conducted across 50 of the 77 EmpaTeach groups by IRC as part of its intervention monitoring activities. During these spot-checks independent auditors observed participants and filled out a checklist documenting timeliness, session coordination, engagement and interactions among participants.
