# Supplementary material for: The EmpaTeach intervention for reducing physical violence from teachers to students in Nyarugusu Refugee Camp: A cluster-randomised controlled trial
Source: PLoS Med. 2021 Oct 4;18(10):e1003808. doi: 10.1371/journal.pmed.1003808 (PMC8489723; doi:10.1371/journal.pmed.1003808)
Supplement: S4 Table — (DOCX) [file pmed.1003808.s007.docx]

S4 Table. Subgroup analyses

| **Subgroup** | **Subgroup size** | | **Adjusted risk ratio in subgroup*** | **Interaction p-value** |
| --- | --- | --- | --- | --- |
|  | **Schools** | **Students** |  |  |
| **Student sex** |  |  |  |  |
| Male | 27 | 826 | 0.88 | 0.528 |
| Female | 27 | 790 | 0.93 |  |
| **Functional difficulty** |  |  |  |  |
| No | 27 | 1,029 | 0.88 | 0.465 |
| Yes | 27 | 587 | 0.95 |  |
| **School nationality** |  |  |  |  |
| Burundian | 10 | 596 | 0.93 | 0.766 |
| Congolese | 17 | 1,020 | 0.89 |  |
| **School stage** |  |  |  |  |
| Primary | 21 | 1,260 | 0.88 | 0.557 |
| Secondary | 6 | 356 | 1.15 |  |
| **School baseline physical violence**** |  |  |  |  |
| Low | 12 | 721 | 0.89 | 0.638*** |
| High | 15 | 895 | 0.94 |  |

* Risk ratio within subgroup for primary outcome (physical violence at midline) in intervention schools compared to control schools, adjusted for randomisation strata. ** School-level of physical violence at baseline, split at the median *** p-value= 0.981 when testing for a linear interaction with school-level of physical violence at baseline.
