# Supplementary material for: The EmpaTeach intervention for reducing physical violence from teachers to students in Nyarugusu Refugee Camp: A cluster-randomised controlled trial
Source: PLoS Med. 2021 Oct 4;18(10):e1003808. doi: 10.1371/journal.pmed.1003808 (PMC8489723; doi:10.1371/journal.pmed.1003808)
Supplement: S5 Table — (DOCX) [file pmed.1003808.s008.docx]

S5 Table. Sensitivity analyses

#### Missing data

For the primary outcome (physical violence at midline), 24/1619 students (1.5%) had some incomplete answer. In this sensitivity analysis, different assumptions were made regarding the missing data.

|  | **Control** | | **Intervention** | | **Adjusted** | | |
| --- | --- | --- | --- | --- | --- | --- | --- |
| Sensitivity assumptions | % | n/N | % | n/N | RR | 95% CI | p-value |
| 1 – All missing experienced PV | 54.3 | 423/779 | 49.8 | 418/840 | 0.91 | [0.81 to 1.02] | 0.108 |
| 2 – Best-case | 54.3 | 423/779 | 48.6 | 408/840 | 0.89 | [0.79 to 1.00] | 0.054 |
| 3 – Worst-case | 52.9 | 412/779 | 49.8 | 418/840 | 0.93 | [0.83 to 1.04] | 0.212 |

Notes: PV=Physical violence, CI=Confidence Interval Best-case: missing assumed not to have experienced PV in intervention group, but having experienced PV in control group. Worst-case: missing assumed not to have experienced PV in control group, but having experienced PV in intervention group.

####

#### Comparison of mean MFQ (depression symptoms):

|  | **Control** | | **Intervention** | | **Adjusted** | | |
| --- | --- | --- | --- | --- | --- | --- | --- |
|  | Mean | (SD) | Mean | (SD) | Diff. | 95% CI | p-value |
| MFQ score |  |  |  |  |  |  |  |
| Midline | 4.53 | (5.44) | 5.57 | (5.27) | 0.07 | [-0.41 to 0.55] | 0.785 |
| Endline | 3.88 | (5.00) | 4.11 | (5.42) | 0.25 | [-0.40 to 0.90] | 0.458 |

Notes: MFQ= “Mood and Feeling questionnaire”
